# Growth factors in orthopedic surgery

**Published:** 2010-02-25

**Authors:** C Zaharia, M Niculescu, N Despa, M Simionescu, V Jinga, I Fleseriu

**Affiliations:** * Titu Maiorescu University, BucharestRomania; ** Colentina Clinical Hospital, BucharestRomania; *** N. Simionescu Institute of Cellular Biology and Pathology , BucharestRomania

**Keywords:** growth factors, stem cells, osteogenesis, osteoconduction, osteoinduction, osteoprogenitor, humoral factors, bone marrow, osteoporosis

## Abstract

Growth factors have represented an essential issue of interest for the researchers and clinicians in orthopedics and trauma over the last 40 years. In the last 10 to 15 years, the advances registered in this field have permitted the identification of the most active cellular and humoral factors as well as the improvement of their use in the orthopedic and trauma surgery. Their domain of application has been continuously enlarged and the results have been visible from the beginning. The authors present their appreciation on the actual state of this subject as well as their experience with results and related conclusions.

## Introduction

For several decades, orthopedic surgeons have been searching feverishly for a new approach in the therapy of the pseudarthrosis, osteochondronecrosis, essential osteolysis or, generally speaking, to treat various diseases that require bone reconstruction.[1,2]

Eclectic, even since the last century, the utilization of the bone marrow, amnios, placenta, bladder mucosa of the human fetus has demonstrated the osteogenic stimulative effect of these anatomical formations that are of mesenchymal provenience. [2, 5]Hematologists have used the bone medullar implants in different blood diseases, with the intention of normal regeneration of blood figurative elements from the original sanguine cell  (‘the root’ where the general terminology ‘STEM’ cell is coming from), and the method has been extended.[3,4,7]

The metaplasic potential of the primordial mesenchyme is not contested, but taking into consideration the bone case (braditrophyc tissue par excellence), besides bone grafts harvested from certain areas (postero–superior iliac crest, ribs etc.), the experience with favorable follow–up, there are some other natural humoral or induced factors added, that are in favor of developing osteoprogenitor factors activity of these grafts. 

Without mentioning the things already known, we will present a few historical facts belonging to this new reconstructive therapeutical thinking, in orthopedics and trauma.

The facts and conclusions we already have, led us to more profound specific studies, in order to identify all stimulative factors of growth and osteogenesis that could be used by the specialists as one of their therapeutic methods.[11,12,25,29]

## General data

Basically, bone–stimulative therapy with growth factors is based today on a few precise findings experimentally confirmed:

1. The common origin (STEM = root) of the cells with great regeneration potential (red bone marrow, granulation conjunctive tissue–young and non–differentiated, vascular endothelium etc.) is mesenchymal.[20,27,30]
2. Great metaplasic and progenitor potential of these cells, with clear trophic characteristics and embryonic origin, which are especially present in the braditrophic structures (as the bone is).[5,6]
3. The growing enzymatic equipment (TGF), the morphogenetic proteins (BMP) and tissular cytokines released in certain conditions are incontestable agents in the stimulation of the regeneration and specific cellular growth with stimulative effect on the functions of these cells.[14,19,24,31]
4. The origin of the growth factors (5 subtypes, among which TGF is the most powerful) is variable enough:
  - Platelets
  - Osteocytes–osteoblasts
  - Bone matrix (IGF Ⅲ)
  - Some fibroblasts (especially medullary endosteal).
  - Medullocytes. The role of the bone marrow in osteogenesis has been underlined since 1877 (Olier), 1881 (Kolliker), 1928 (Sandison) and 1934 (McGrow). Nade, Burwell (1977) and Salama (1983) have developed the researches by applying the observations to the clinical backgrounds (Ghergulescu, Diaconescu and Zaharia in our country)
  - Some components of the deep layers of the periosteum (?)
  - Humoral products derived from clot lysis phases (?)

Experimentally, it has been demonstrated that mammals have molecular signals and reactive cells that initiate osteogenesis in their bones. These cells have two precursor cellular lines: one determinant line and one inductive line. The cells derived from the last one are responsible for the bone formation, through induction, by demineralized matrix (extracellular) and morphogenetic bone proteins. Recently, the BMP genes have been cloned and the modality of action of the recombinant proteins has been clarified:  pleiotropic initiators of the precursor inductive osteogenic cells.[13,30,31,34]

Osteogenesis is governed by the BMP in all three stages:

1. chemotaxis
2. mitosis
3. differentiation

Recently, the BMP receptors have been identified and cloned, they are of two types (type Ⅰ and Ⅱ), divided into membranes–limit seryn/threonine ‘proteinkinase’. With the help of these receptors ‘the binding’ of two extracellular matrices is achieved and thus, ‘continuing and rhythmic differentiation osteoblast–osteocyte’ is determined.[20, 24]

From the things shortly exposed above it is easy to understand why the researchers are so concerned about using BMP in clinical cases.

Jensen, Overgaard, Lind (1998) have highlighted the importance of the osteogenic protein OP–1 (BMP–7) in osteoinduction, this protein having a determinant role in the bone graft fixation at the site of the receptor host. This protein action manifests on the collagen fibers, on the hydroxyapatite and on the matrix of the minerals fixation (stimulative role through osteoinductive effect that has as a result the ‘capturing’ tendency of these matrixes, of the stem cells and other osteoprogenitor factors).

These few conclusions represent only the essence of some long time research in the  domain; right now we are in the phase when the eclectic approach is leading us towards certain directions (let's remember the 60–70 years when preparates of the placenta, such as the amnios, fetus bladder mucosa, even staphylococcal anatoxin, were very well used in the local treatment of pseudarthrosis).[9, 10, 16, 32]

## Bone reconstruction mechanisms

The basic biological processes that lead to bone reconstruction, a domain we cannot act by stimulating them, are osteoconduction and osteoinduction.[29]

**Osteoconduction** is represented by the formation and the development of the conjunctive–vascular buds and those of the adjacent structures (of mesenchymal origin) that grow and penetrate the tissues and are repopulated with the help of local osteoprogenitor cells or with cells derived from autologous cultures. The process of participation of the osteoprogenitor fibroblasts from the homologous cultures to osteoconduction, into bone grafts sites is still being studied. Certainly, osteoconduction is efficient only if both osteoinductive substances and osteogenic cyto–humoral elements are present in the same region. Among the allogeneic materials (substances) that were credited with such properties only the Calcium sulfate, bio–active glass and some polymers (still in experimental phase) have given encouraging results, the others (hydroxyapatite, phosphates, aluminum derivates) have offered contradictory results (clinical and experimental). These materials have an important helping role in the treatment of large bone defects or of big floating pseudarthrosis, in association with autologous biological materials (spongious auto–grafts, preserved depopulated matrix, medullary aspirate) that have an osteoinductive role.[21,28,37]

**Osteoinduction** represents the totality of the humoral factors, physical, cellular (primordial) and of chemotaxis (probable), which by their local and general action they influence:

1. The production and complex adjusting (hormonal, biochemical, trophical) of the enzymatic osteostimulatory factors.
2. ‘The calling’ in and at the site, of the mesenchymal cellular elements with osteoformative metaplasic potential (cells with ‘adult’ STEM cell characteristics), simultaneously with the synthesis and the increase of the local concentration of some enzymatic factors (growth factors) with inductive role.

We remembered the most well known factors (TGF–beta1–2, BMP5, the complex BMC–BMP, BBC–BMP etc.) besides the physical procedures (electrical and electromagnetic, ultrasounds etc.), but the research continues for the identification of certain substances that appear during bone remodeling and reconstruction process, with an obvious action (we remember our research in osteoinduction activation after the lysis phase of the clot).

The theories of the osteoconduction can be sketched, as evolution in time as:

1. Induction through BMP (Wrist, 1967).
2. Metaplasia of the yellow bone marrow into osteoprogenitor tissue when it is transferred into vascular zone. (Tavasili, 1970)
3. Theory of the double origin of the bone callus (Dan,1973)
4. Induction by BMP and other humoral factors (Linholm, Wrist, 1980–1987)

We have to take into consideration the osteoinductive mechanisms in the osteogenesis stimulation and structural formation, in function of the mechanical tasks from a bone modeling or remodeling area, in order to influence them, eventually.

The osteoinduction, a notion that includes local and general mechanisms with biochemical characteristics, enzymatic, physical and of tactism, determines:

1. The selection of the cells of mesenchymal origin with multiple metaplasic potential (STEM), but which can be stimulated into osteoformative direction.
2. The production and contribution of the growing humoral factors (osteoprogenitors), from the humors of the body, own bone matrix, local enzymatic reactions (clot lysis), the cells and sanguine protein factors, etc.
3. Local neuro–vasculo–humoral adjusting through some tissular receptors (hypothetical) of the complex process of osteolysis–bone reconstruction (osteoclast–osteoblast activity).
4. The changes of type A collagen, following the conduction, in the early stages, of the proteoglican synthesis (agrecan–organizer of the extracellular matrix) by several factors, in such way that the new–formed bone suffered variations into its composition until its definitive structure was complete (osteoinduction initiation would be started by an osteogenic protein–OP1 –after Jensen, Overgaard, Lind and Wrist).

To the three initial classic groups of growth factors (TGF–b) represented by the BMP group (11–12 factors), TGF–b group (5 factors) and the group of the inhibins (3 factors), we have added (after clinical observations) and researches another 4^th^ group, of local tissular stimulins (cytokines) which appeared from the clot lysis and from local destructions of the tissue.

Regarding the fresh spongious bone graft that contains mesenchymal STEM cells type, which present a combination of the osteoinduction process with another plastic complex process, osteoconduction, during its integration, it assures a balanced three–dimensional remodeling of the new bone on the offered ‘skeleton’. Osteoblast activity is situated in the center of the osteoconduction mechanisms. Moreover, it is implied in all the phases of this mechanism such as ‘the quiet period’, ‘the activation period’, ‘resorption’, ‘the reversion period’ and ‘the new bone formation period’.[15]

## The growth (osteoprogenitor) factors diversity

Cellular factors:

- STEM type cells–medullocytes, undifferentiated fibroblasts
- PDGF, resulted from platelets
- Osteoprotegerin from osteoblasts–with action on the RANK–receptors (bone resorption inhibition)
- Sinoviocytes that in certain conditions modify their metabolism and become cells with osteoclast characteristics
- Substances from the medium culture that are inducing the increased activity of the phosphatases

The main source of stem cells is the embryo (embryonic cells), with multiple potential and which can be preserved for a long time. In order to obtain cells with osteoprogenitory capacity from the adult, these must be harvested from the foam desmale bone (iliac crest, rib, vertebral body), which has the largest medulocytes concentration and has the ability to form fibroblasts with osteoformative potential (CFV–F)[1, 2, 3, 4, 5]

Humoral factors:

- TGF (5–6 subtypes)
- BMP
- Tissular cytokines
- Prostaglandins
- Insulin Like Growth Factor (IGF)
- Fibroblastic Growth Factor (FGF)
- VGF
- Epidermal Growth Factor (EGF)
- Interleukins (I1–1, I1–2, I1–10)
- Different forms of eritropoetins
- Substances derived from the sanguine clot lysis

Other factors:

- Factors of the bone matrix:
  - desmal bone matrix – with osteoinductive action
  - pleiotrophins from the bone matrix
- Chemical factors – dexamethasone – in vivo
- Metals: Zn, Ti
- Electrical factors – galvanic fields

Changes that reveal the fact that bone remodeling is happening in small morfo–bio–chemical packages, coherent, rhythmic and harmoniously coordinated can be observed at the level of the general skeletal homeostasis, during the bone growth stimulation process (Parfitt, 1988).

Different enzymes, cytokines, humoral factors with multiple potential (prostaglandins, calcitonin) interfere in different osteogenesis phases in the bone remodeling sites, graft integration, osteolysis etc., and the processes are unfurled at the level of the so called ‘bone remodeling units’, in time units determined by the local resorption–reconstruction process (bone turn–over rate), irrespective of the bone remodeling modality. (woven–bone or ‘direct’ remodeling).

It is known that, in certain conditions, the cells from hematopoetic stromal medium selectively interact with unspecific STEM cells and influence their development (experiments on mouse), even if these are not coming from hematopoetic STEM cells, this demonstrating the very great plastic potential of the STEM cells.

The actions of some factors like the following are still being researched today:

- ‘Alpha’ (TGF) Growth factor  from tumoral cells culture
- Tumoral growth factors, would be of interest if they could be controlled from the quality and quantity point of view
- Transforming growth factor beta (TGF–beta)
- Insulin–like growth factor (IGF)
- Fibroblast and epidermal growth factors
- Some of the interleukins
- Eritropoetin
- Piezoelectricity activity and ‘narrowing’ the potentials (streanning) of the fluids to flow into the bone
- Some metals (titan, zirconium)
- ‘Anchoring’cells
- Some of the proteases (metaloproteases);
- Depopulated bone matrices, degreased, dried and sprayed, with an obvious osteoprogenitor role in the case of their utilization as autologous graft or added in the medium culture of the autologous osteoformators fibroblasts
- The interferon
- Some retroviruses
- Some markers or substances appeared into the osteoblasts' medium cultures (sialoproteins, osteoprotegerin, etc.)
- Sinoviocytes (as mesenchymal cells with different potential)
- Some substances (dexamethasone, calcium preparations, intermediar products of Vitamin D3 metabolism) that positively influence the osteoblasts' cultures

## Practical utilization of the osteoprogenitor factors

The history of the use and the research of the STEM–type preparations is unfurled on a long period of time (about 35–40 years), from simple clinical and statistical observations reaching today, in the phase of synthesizing specific proteins and enhancing cell cultures.

We can affirm that the bases of the systematic study of the STEM cells' role into bone consolidation belong to Maureen Owen (1976, 1988, 1995), but earlier to Wrist (1965–the first who mentions the existence of BMP into osteoblast). Raisz, Rodan, Creenshaw, Frost, Yamaguchi, Ripamonti, V.Ranga etc. have contributed to this study.

Recently (1977), in our country, N. Ghergulescu and S. Diaconescu, in Republic Moldova V. Betisor and collaborators, then  Kirehead, Reddi, Jefferson, etc., have enriched the clinical studies in this domain. In ‘Colentina’ Clinical Hospital from Bucharest we are concerned to improve the application in clinical activity and to research other STEM cells sources, as well as bone progenitor preparations (from 1978), having a significant number of cases and a wide range of applications currently available.[17,35]

The last years before the interesting discoveries and the role of some growth factors, not far behind in a time only ߢguessed’ as having osteoprogenitor activity, the following have been clarified:

- TGF (Wrist)
- BMP (Beck)
- STEM replication factors
- ‘Selectioned’ cell cultures (Roberts)
- Obtaining and maintaining some mesenchymal cell lines with high osteoprogenitor potential (C3H1OT1/2–Weber) in laboratory conditions

## Cell cultures

In the laboratories of ‘Nicolae Simionescu’ Institute of Biology and Cell Pathology  from Bucharest, which we collaborate in the field of basic and clinical research with, we managed to obtain proliferation, differentiation and ‘characterisation’ of the osteoblasts from mesenchymal cells isolated from human bone marrow, after a very sustained activity of medium cultures preparation and after obtaining the means to preserve the cultures. Therefore, today we are able to use these cultures in surgery practice (sustained by different organic or synthetic supports, with different structures).[1,2,5,10,32,33]

## Materials and methods

**Fig 1:**
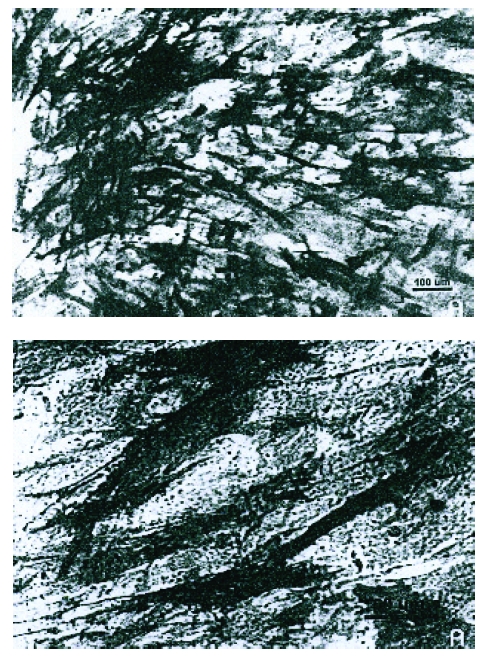
Making evident the alkaline phosphatase in osteoprogenitor cell cultures from spongy bone explant  (‘N. Simionescu’ Institute of Cellular Biology and Pathology).

From the bone marrow samples of different human patients, harvested by puncture from the postero–superior iliac spine, nucleotic cells have been separated on a density gradient and they have been grown in DMEM 0.45% with a supplement of 15% fetal cow serum (SFB) and 10 mg/l of ascorbic acid. The osteoprogenitor cells (CO) obtained, are characterized by ‘fibroblast like’ morphology and a positive reaction to alkaline phosphatase. The following implant materials have been used: pancol (P), matrix MG (MG), spongious human bone without cells and different hydroxyapatite (HA). The implant materials have been conditioned in the same environment. We have highlighted that the cells have populated these implants through different optical and electronic microscopy techniques. CO differentiation into osteoblasts has been tested by putting in evidence the specific markers as: osteonectin (Osn) and bone sialoprotein (BSP) through immunofluorescence techniques.[Fig 1]

A number of enzymes (alkaline phosphatase, osteocalcin) highlighted in fibroblast cultures, along with qualitative determination of sialoproteins are the first signs of conversion towards osteoformative cells of the mesenchymal cells elements.

**Results:** Studies of optical and fluorescence microscopy have shown the various degrees of cellular population on the implant materials and HA used. A better settlement has occurred on Pancol and between three–dimensional tested matrices, the best populated has been the human spongious bone. In the HA case, the factor that has determined the adhesion and cell multiplication has been the crystalline structure of the used hydroxyapatite. The ultrastructural examination (electronic transmission microscopy) has tested the presence of a rough well developed endoplasmatic reticle (RER), a great number of free ribosoms and secondary lysosoms in the case of matrix integrated cells. The reaction to alkaline phosphatase has been followed to see if it remains positive to cells grown on the HA coated supports. The immunofluorescence techniques have demonstrated the presence of Osn and BSP in the cells grown for one week on HA matrices.

**Conclusions:** In the case of implant materials, the best settlement is achieved on the depopulated human spongious bone. Even in the cases in which the re–population of the implant matrices is reduced, no ultra–structural changes of the cells were registered. On the tested HA matrices, the cultures show very few differences regarding the alkaline phosphatase quantity and the differentiation between osteoblasts is not realized in all cases. The implants populated with human CO are used as auto–transplants in Orthopedic and Trauma Clinic of ‘Colentina’ Clinical Hospital.[23, 35]

**Technique and methods** of using the preparations are varied and some authors searched for ways to standardize them. This thing is still impossible as long as the role of some factors in certain phases of the osteogenesis, is not yet clarified.

This is the reason why we expose the methodology used by us, with personal contributions to the clinical practice.

**Fig 2:**
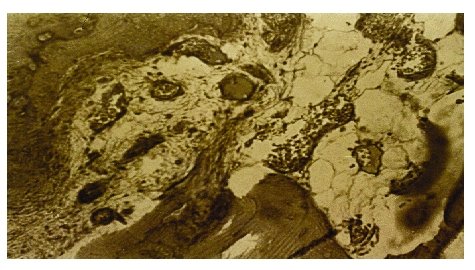
Bone graft with bone marrow aspirate from SIPS (center) (microscopic appearance, x 40, Van Gieson)

1. The massive bone graft containing STEM cells is usually harvested from postero–superior iliac crest (SIPS); this can be autogenous (the best situation) or homologous (with recommended tests done). We are using this ‘on–lay’ or ‘in–lay’ in lax pseudarthrosis infected or not infected, in great lacks of bone substance (essential osteolysis, osteitis with great volume of sechestrum elimination, bone defects after complex trauma with bone substance losses). Dysplastic diseases of the long bones or the osteochondronecrosis (NACF, knee osteochondritis), as well as infected pseudarthrosis of the mandible are lesions that have this method as treatment indication. [Fig 2][23]

The osteomuscular decortication in pseudarthrosis, is an excellent adjoin; besides its known qualities, it is also added the finding that this ‘guided comminutive fracture’ contributes to the releasing of osteoprogenitor cytokines. In the cases of great lack of bone substance, we have used the combination of osteoprogenitor graft (SIPS, ribs) with great fragments of ‘depopulated’ and despecified bone. This has been realized by conservation to –4 degrees C, in a sterile solution (culture medium for vegetal cells) type B5 (Gramberg), rich in mineral salts and some organic substances (zaharose, vitamin B) and a constant Ph (6.83–6.85) for a very long time (6–8 months). Only the bone matrix of these grafts is kept strong, solid and well mineralised and the cells of any type, yellow marrow and soluble proteins disappear. Implanted at the pseudarthrosis site, very well prepared before and adding alive preparations of STEM type (grafts, macerate, and suspensions), the reconstruction is fast and firmly. The graft's re–population is done in a short time and on the old architectonic structures or other new ones, depending on the mechanical line forces. For the NACF, after practicing a trochantero–capital tunnel of about 4–8 mm diameter (hand drilling) under Rx–TV control, the STEM preparation is inserted with the help of a calibrated trocar. The STEM preparation has a form of ‘carota of drill’, harvested from SIPS, without doing the curettage of the NACF site (we are also repeating an earlier finding: NACF does not mean necrobiosis but coexistence of an anarchic destruction–reconstruction process, due to localized vascular and mechanical disorders; besides, this drilling process that generates the release of local cytokines, that induce (through a similar mechanism to that exerted by interleukin–6) the bone reconstruction processes through the game of ‘reciprocal induction’ between osteoblast and osteoclast.

2. **STEM preparations**. They consist of selected soak spongious bone (SIPS, ribs) or soak STEM bone mixed with SIPS aspiration product, resulting in a kind of paste easy to be injected with the help of the trocar or easy to be modeled in a cavity zone. We are not recommending heparin to be added for stopping blood clot formation, just because the simple agitation of the preparation before being injected is enough to make it fluid, the blood clot being important through the osteoprogenitor factors released in the moment of the clot lysis. These osteoprogenitor factors together with graft medullocytes have an important role in osteogenesis (tixotropy).[Fig 3]

**Fig 3:**
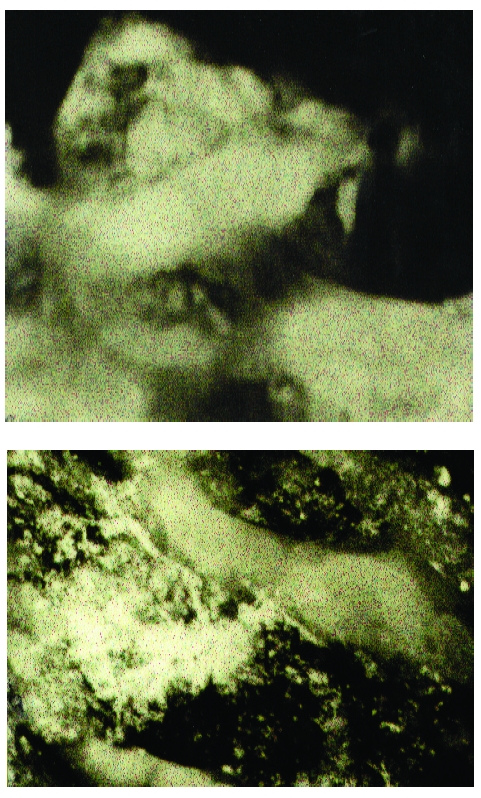
a. Autotransplant, without instillation of osteoprogenitor preparation in the site, 10 days after the implantation (dog experiment, tetracycline fluorescence x 100). b. Similar case which was added preparation. Note the intense activity of cellular repopulation.
3. **Mix implants** (fresh graft from SIPS + depopulated graft + STEM type soak). They are used in great lacks of bone substance (pseudarthrosis and essential osteolysis or pseudarthrosis of the long bones appeared post–trauma, elongations of the long bones that have a small osteogenic potential – humerus, tibia, etc.)
4. **SIPS osteocartilaginous graft**. Harvested from child or adolescent it is used in NACF or knee osteochondritis. For this aim, it is shaped in a ‘carote’ form, from SIPS, to size lesion, 15–20 mm long (to reach the vascular region) and the spongious bone adjacent to the articular cartilage is removed. Thus, the graft is implanted with the cartilage toward articular cavity (on the trocar) for femoral head or through arthrotomy for the knee.[18,26]
5. **A personal procedure**. Spongious bone implant fragmented or soak from the osteogenic exostosis of children or adolescents have been used with excellent results. Of course, these exostosis have undergone anatomo–pathological examinations and analysis required as homologous grafts (HIV, AgB, C etc.). We found that these exostosis, especially in some phases of growth, are extremely rich in medullocytes and in the regions (laminaes) of the new young bone, they are organized in Haversian lamelar systems, very well evidenced by PAS staining.[Fig 4,Fig 5,Fig 6)

**Fig 4:**
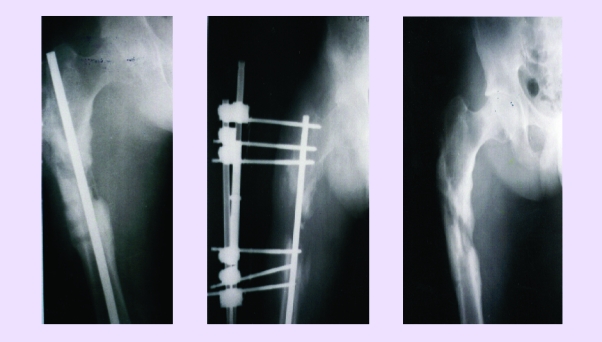
a. Secondary pseudarthrosis after osteitis of the femur, with large loss of bone capital–mixed fixing after the drain of the site. b. 60 days after grafting and instillation (every 14 days) of osteoprogenitor preparation. c. The same case, 5 months later.

**Fig 5:**
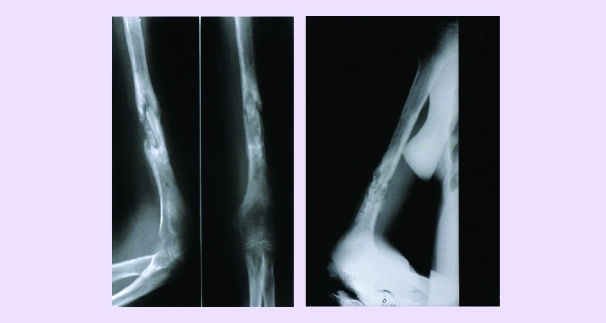
a. Infected pseudarthrosis (osteitis) of the humerus. b. 3 months after local treatment (instillations of osteoprogenitor preparation) at every 21 days

**Fig 6:**
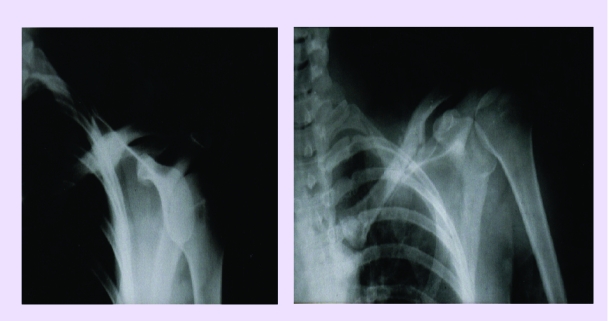
a. Pseudarthrosis of the clavicle. b. The same treatment as in Fig 5, 60 days later.

**Casuistic:** Our experience spans the period 1978–2008 and compiles:

1. Pseudarthrosis (52 cases):[26]
  - clavicle (infected and non–infected)
  - femur
  - tibia
  - humerus
  - intertrohanterian varisation osteotomy sites
2. Avascular Femoral Head Necrosis (48 cases):[17,18]
  - idiopathic
  - secondary
3. Osteoarthritis (knee osteoarthritis, hip osteoarthritis) (89 cases)
4. Post–traumatic osteolysis (5 cases):[22]
  - clavicle
  - femur
  - humerus
5. Essential osteolysis (idiopathic) (2 cases)
6. Disecant knee osteocondritis (3 cases):
7. Limb elongations (6 cases):[Fig 26]
  - femur
  - shank
  - humerus
8. ‘Stress’ osteoporosis (11 cases)[Fig 38]. This entity has been individualized and studied by us, especially in the last 14 years. There are local osteoporosis (postoperative, generally), regional, painful, embarrassing, that determine a hard mobilization of the patient. Our cases treated through STEM type grafts into the greater trochanter region or into the proximal methaphysis of the femur are:
  - intertrohanterian osteotomies
  - cervico–cefalic prosthesis
  - total hip cemented prosthesis
  - humeral trohiter in a case of very painful PSH
9. Secondary osteoporosis
  In the case of pseudarthrosis [1], our surgical treatment method can be outlined as it follows:
  - drills and minimal guided fracturing in the pathological bone
  - instillation with a special syringe of a mixture containing factors with osteogenetic stimulative action, clinically and experimentally confirmed by our researches

In the surgical interventions for osteoarthritis [3] a special place is reserved to osteotomies, as well as to other procedures, as alternatives to the total knee prosthesis.[15,35]

Our personal casuistic is made up of a number of 80–knee osteoarthritis operated by other surgical procedures than the total arthroplasty, followed up between 2 and 10 years. General conclusions are especially discussed in light of the results of some non-invasive procedures used by us. After that, we have observed encouraging favorable follow–up for long periods of time (subcortical spongioclazia, trophic drills etc.). Starting from these observations (into the cases of painful knee osteoarthritis) we have extended the methods to hip osteoarthritis and finally, we have presented some conclusions that are recommending the use of this procedure (spongioclazia).

There were also situations when major surgery (total prosthesis, the osteotomy) could not be practiced, the patient requesting the improvement of his condition (pain, the decrease in the movement's amplitude, difficulties in walking). In these cases we have practiced a less invasive procedure (spongioclasia), presented below.

**Fig 7:**
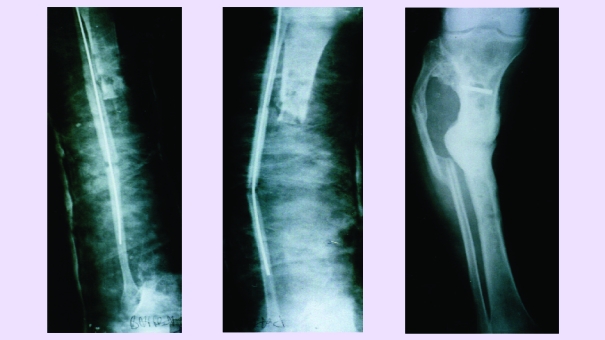
a. Open fracture, with large loss of bone substance (shank); b. The same case 6 months later (autografts with homolateral fibula and instillations at every 21 days).

**Fig 8:**
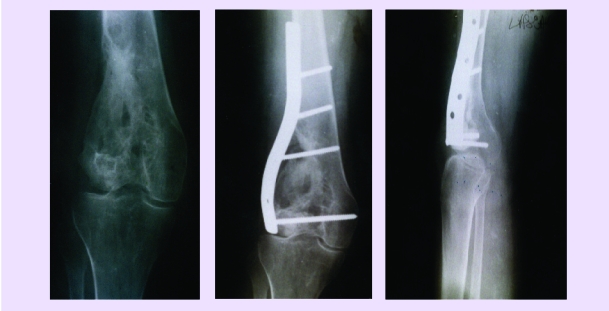
a. Resection of the medial condyle of the femur for giant cell tumor – osteoplastia with iliac bone and patella; b. Reconstruction and perfect reintegration 4 months later, during this time are made instillations every at 30 days.

**Fig 9:**
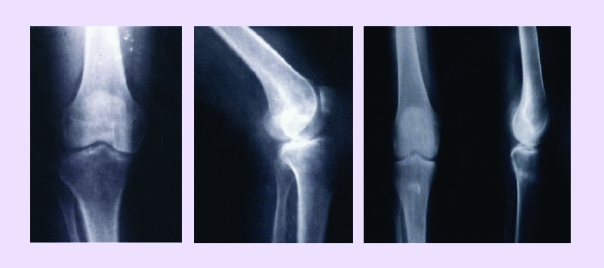
a. Idiopathic knee arthrosis; b. Same case, 3 months after spongioclasia and instillation.

The main indications for this type of surgery are:

- Different associated diagnosed diseases that established a contraindication for a major surgery
- Obesity, that patients could not or did not want to treat.
- The patients' refusal to undergo a major surgery with a long period of recovery, from different reasons (fear, the lack of an assisting person, the impossibility to interrupt professional duties, etc.)
- The possibility to recover very quickly and be back to the quotidian activities and the advantage of having the recovery treatment in ambulatory conditions. [Fig 7, Fig 8, Fig 9]

**Fig 10:**
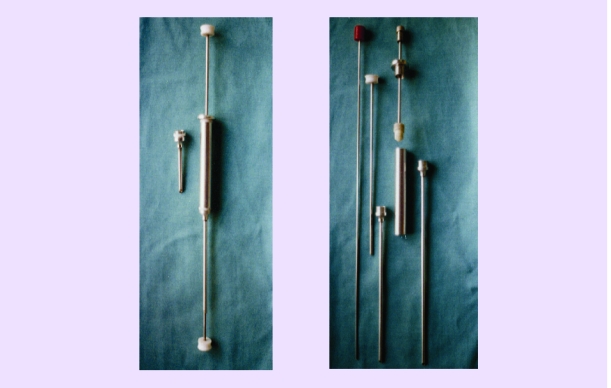
Syringe for instillation

**The surgical technique** [36,38,39]to the knee level is relatively simple. After marking the important anatomical reference points (tibial tuberosity, femuro–tibial interarticular line, the head and the apex of the fibula, the anterior crest of the tibia and the postero–medial edge of the tibia) on the skin, an oblique line will be drawn beginning from the postero–medial edge of the tibia (corresponding to tibial tuberosity as a landmark) which passes in the immediate proximity of tibial tuberosity and ends at the fibular apex. Through a vertical incision of 0.5–2cm (in the case we are also introducing a bone graft) or through a simple hole prevailing on the medial region of the shaft on this line, the tibia is approached at 15–20 mm distance ventral of the postero–medial edge. [Fig 10]

**Fig 11:**
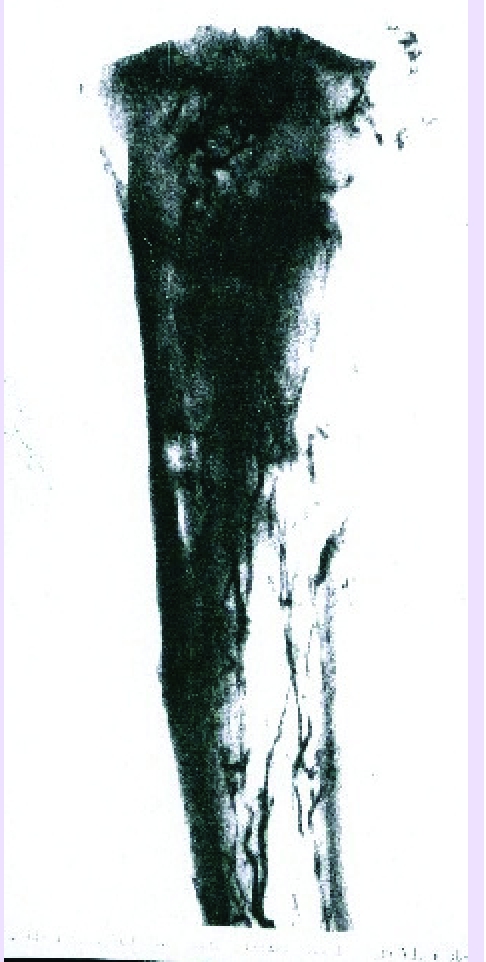
Angiography of the proximal extremity of the tibia. Note the diaphyseal arterial circulation proceeded from the nutritional artery, and the epiphyseal one from periostic and capsulo–ligament sources.

A hole in the cortical is done with a manual drill (crank, chisel of 8 mm) and a blunt instrument is introduced (spongiotom) by sectioning the spongious bone in a circular manner, always following the internal contour of the cortical bone. This way we obtain a subcortical ‘osteotomy’ of the spongious epiphyso–metaphysial structure (subcortical spongioclasia). In addition, we partially obtain a combination of the osteotomy and the drill effects. In cases of more advanced osteoarthritis (std. Ⅱ, Ⅲ and even Ⅳ) where the subchondral osteonecrotic lesions at the medial condyle level are constant, we introduce a cortical graft (autologous or homologous) 3–5cm long, placed transversely, so that anterior and posterior communication exists, with the cavity created during spongioclasia, for the venous drainage. The trophic and bone regenerative effect of this graft, observed much earlier and subsequently confirmed is also completed by our observation that its integration as well as the remodeling of the bone are ‘sustaining’ elements of the tibial condyle. Therefore, the deformation and tibial varisation are stopped or even reduced.[Fig 11–Fig 13] 

**Fig 12:**
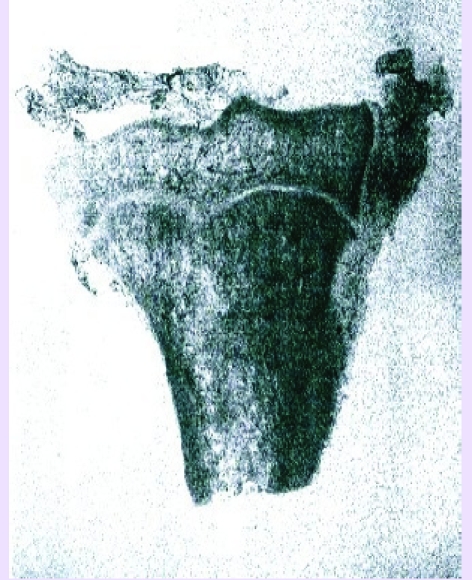
The two territories with nutritive irrigation are separated at the level of former growth cartilage.

**Fig 13:**
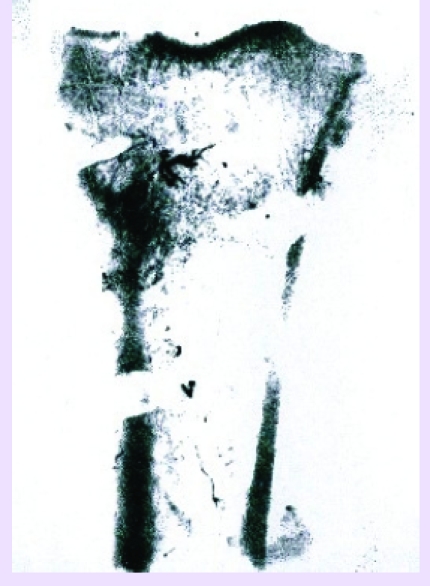
After spongioclasia (3 months) an improvement of the arterial epiphyseal irrigation (experimental, dog tibia) is noted.

During the last 5 years, we have stopped performing bone graft procedures while we continued performing only spongioclasia.

We have instilled an autologous preparation (STEM osteoprogenitor) in the site of the spongioclasia, whose short–term (2 years) effects are more than encouraging.

In the last 4 years, we have added the perforation of 4 channels in the metaphysial plaque (the old growing cartilage) to assure a circulatory diaphysial medulo–endostic flux towards epiphysis, which, in the case of the tibia, is predominantly of a periostic or capsulo–ligamentar origin.

After the operation, walking debuts in 24 hours using cane or crutches (50% load for 14–21 days), continuing with the progressive resumption of full support in 35 days. From the 21^st^ day, the patient can begin physiotherapy (profound diathermy, ultrasounds) and muscular massage. This recovery treatment is repeated 2–3 times per year, for up to 3 years.

Sex distribution of patients point out that women have registered higher scores (postoperatively) than men (the same procedure to the hip level seems to give better results in men).

From the age point of view, the best results have been encountered in the 50–65 year old segment of age, poorer results over 65 y.o. and partially good (especially in pain improvement) in the 35–60 years range. In some patients with painful sechelae we have associated articulary denervation (4 cases) followed by a durable improvement.

**Fig 14:**
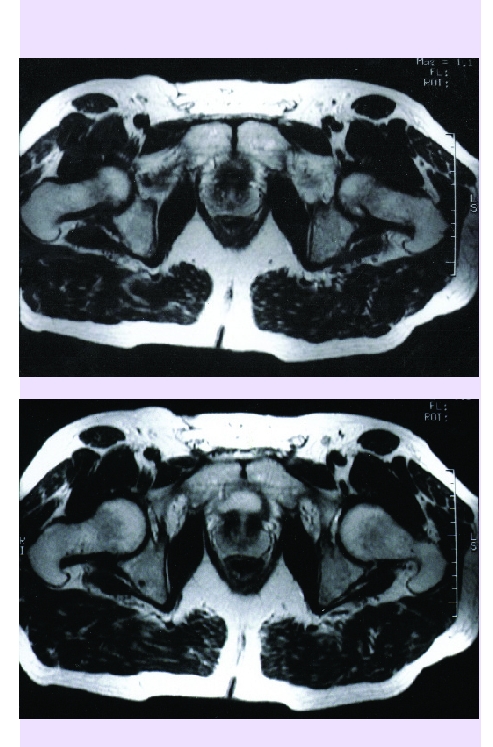
MRI before (a) and after (b) spongioclasia and instillation of preparation (hip arthrosis) at every 3 months.

All the cases that have been treated through spongioclasia have benefited from physio–kineto–balneotherapy, that undoubtedly contributed to the quality of results.[Fig 14]

**Conclusions:**

**Fig 15:**
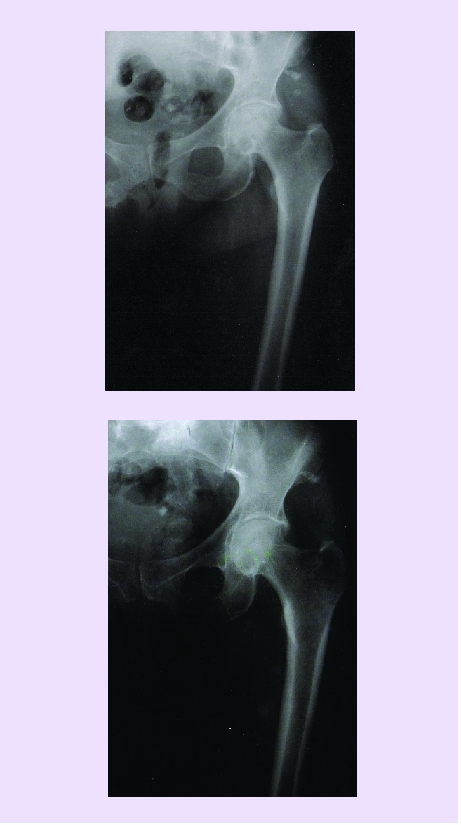
NACF, before (a) and after (b) instillation of osteoprogenitor preparation by trochantero–cervico–capital drilling (result after 3 months).

**Fig 16:**
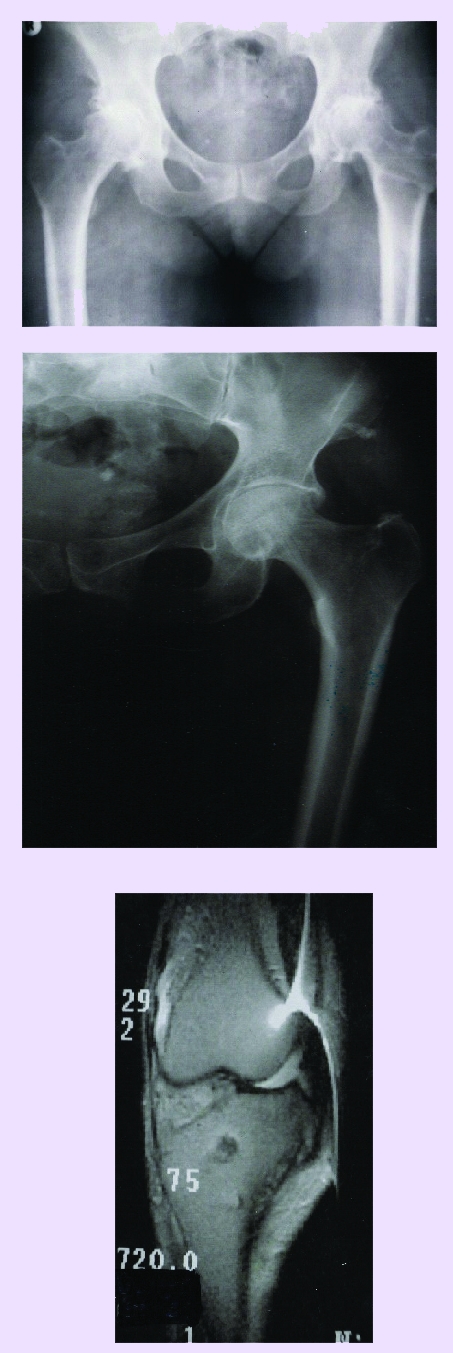
Similar case, the same treatment. Initial appearance (a) and 3 months later (b)

**Fig 17:**
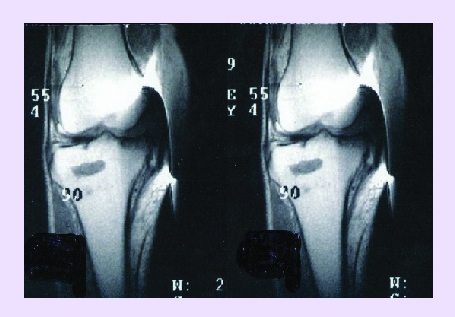
Idiopatic gonarthrosis immediately after spongioclasia and stem instillation (a) and 3 month later (b).

- this surgical procedure is minimally invasive and very easily performed
- surgical procedure ‘of waiting’, (very often with definitive character or for a long period of time) if the patients follow the postoperative treatment very accurate
- Regional trophic circulation is improved, with remarkable results regarding pain and the functionality of the knee, probable due to:
  - use of autografts and homografts, biological or synthetic matrices, treated with osteoprogenitor factors associated with osteoblasts cultures
  - bone remodeling stimulation
  - a new architecture of the spongious bone (similar to a normal one)
  - intra–osseous denervation obtained through spongious bone trituration, with antialgic effect. [Fig 15,Fig 16,Fig 17]

**‘Stress’ osteoporosis** segmental [8] has been recently individualized. In 1994, Gallant, Fisher, Sziklas (Connecticut) have been between the first clinicians who have signaled the post–traumatic regional, post–operative and post–inflammatory osteoporosis (foot, ankle), with a very close simptomatology to that of algic osteoporosis (Leriche). However, the segmental osteoporosis (of a restrained circumscribed zone) that appears isolated at the great trochanter level, acromion, clavicle extremities, distal epiphisis of radius and ulna, lunatum, trohiter, has not been studied or specially evidenced.

Segmental osteoporosis is also mentioned at a vertebra level (Garland, Foulkes, Adkins, Stewart, Yakura –1994) at variable intervals after trauma. Simptomatology and the disease follow–up have identified almost entirely, what we knew as Kummel–Verneuil syndrome (studies 1 and 2).

Several years after the beginning of mainstream metal or ceramic implants (hemiartroplasties, artroplasties or artro–osteoplasties in different diseases – arthrosis, rheumatisms, tumors) an increased number of loco–regional segmental osteoporosis or generalized osteoporosis has been observed.[38

**Fig 18:**
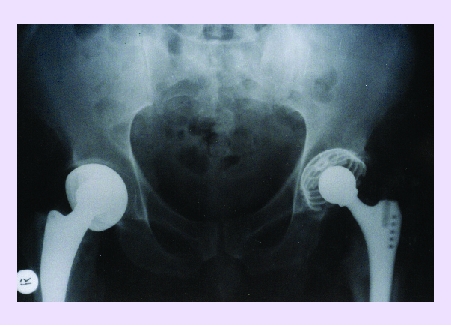
Regional osteoporosis (after bilateral PTS), acetabular and trochanterian.

**Regional segmental osteoporosis** (causalgia, Sudeck osteodistrophy, postraumatic osteoporosis, algodistrophy, shoulder–hand syndrome) has various causes:

- Post–traumatic
- Post–operative
- Post–insertion of an implant:
  - polyethylene
  - cement
  - metallic fragments
  - mechanical stress
  - bone nerve destruction
  - bone vascular destruction.[Fig 18]

Installation mechanisms can be ordered:[15,35]

- Chemical (polyethylene, carbon)
- Local biological (cement fragmentation or detachment of small metallic particles from prosthesis)
- Local congestion determined by the ‘foreign body’ existence
- Local elasticity ‘incompetence’
- High initial temperature of the cement (80 degrees)
- Tissular destructions consecutive to local drills
- New mechanical stress, non–physiological (local) due to changes of original movement axes
- Type of the prosthesis sterilization (best way with Gamma radiation)

In the case of older people, the osteoporosis manifestations appear earlier and intensely because of the osteoclasts and macrophages predilection to activate themselves at minimal stimuli (Kevin D.Freston), but clinically and radiologically, a healthy bone does not appear with big differences in comparison with an operated one, as it was observed in younger patients. Osteodensiometry is the investigation that establishes the positive diagnosis

The installation mechanism of this osteoporosis of ‘prosthesis’ could consist of a stimulation of the unspecific activity of the white blood series, the activity of the osteoclasts being concomitant, but secondary as importance (Howie, Maloney, 1989; Parr, 1995; Lauzer etc.).

This opinion can be sustained by anatomo–pathological observations that are showing, on samples taken from the region, granulomatous lesions with giant cells, histiocytes with phagocytic properties, along with metallic debris, polyethylene debris, ceramic or poliacrylic cement debris.

We cannot still say for sure which type of total prosthesis (hip or knee) cemented or non–cemented, leads to the appearance of local segmental osteoporosis in a greater percent. Regarding the non–cemented prosthesis, Charnley was the first to signal osteoporosis that is still present even 10–15 years after the operation. The same thing is signaled even since the 1970s by Maloney. The incrimination of the cement is also sustained by Harris, Santavirsta etc. Whichever the cause, osteoporosis of this type is extremely refractory to the treatment.

Decades ago, Urist was defining osteoporosis as ‘the most common bone dismetabolic disease’. The modern reality lays this disease close to obesity and diabetes mellitus as the top metabolic diseases, but it is distinguished by the fact that the endocrine and nutritional elements incriminated in its own etiopathogeny cannot be objectively quantified.

Being a chronic disease that interests the skeleton totally or partially, transitory or chronically, the osteoporosis is characterized by anomalies to microscopic and macroscopic level of the bone tissue (having as main characteristic the bone mass reduction as a result of specific matrix alteration) on unit volume of this mass. The consequence of this trophic–metabolic deficit is bone fragility, which can determine the increase of the risk of fractures, para–articular deformities (by trabecular micro–fractures into the spongious epiphysial bone) and often very serious functional perturbations.

Secondary osteoporosis [9] has as main causes the following:

- Systemic immunological diseases (rheumatoid arthritis)
- Osteoporosis caused by effort: young athletes (calcaneum, cuboid, navicular bone)
- Professional osteoporosis–X–radiation exposure, high temperatures in steel works, variations of gravity (astronauts), vibrations exposure, prolonged intellectual stress
- Osteoporosis caused by chemical–biological factors –Pb, Si, Sr, Cd, Hg, cyclic carbohydrate, oligosymptomatic chronic infections
- Osteoporosis caused by medicines–cortisone, AINS, diuretics, immuno–suppressive medicines. This entity is more frequent than we are tempted to believe.

The treatment proposed by us consists into the following surgical procedure:[38]

- Bone aspirate from SIPS that is containing STEM cells (mesenchymal cells)
- Dexamethasone
- Manual lysis of the sanguine clot to release PDGF from platelets (tixotropy)
- Active forms of Vitamin D3

This combination is injected in the osteoporosis site and has as result the diminishing of pain. The patient remains to be followed up and is associated, where appropriate, physiotherapy and kinetotherapy.

Good results obtained in these cases (as well as in the patients included in paragraphs 1, 4 and 5) could have possible explanations like:

- Sanguine clot lysis–release of PDGF
- Dexamethasone–initiates STEM cells transformation
- Proliferative response to PDGF action, which is observed in several types of mesenchymal cells (STEM cells)
- TGFbeta derived, probably from active TH1 cells (T–helper) and from natural–killer (NK) cells and those exert anti–inflammatory effects (suppresses cytokine production), stimulates healing, inhibits macrophages and lymphocytes proliferation

## Conclusions

Discussing the results while taking into consideration an objective ‘score’ is not possible. The cases, even though various, evolving almost identically (as stages), except for some diseases (limb elongations, infected pseudarthrosis, secondary and essential osteolysis) required prolonged time to be solved

Clinical and laboratory means for patient evaluation are revealing, but for interpreting the therapy results with these factors, we recommend assessment methods such as:

- Clinical evaluation:
  - pain
  - mobility
  - different scores
  - subjective element : patient satisfaction
- Paraclinical tests:
  - X–rays
  - MRI
  - CT
  - scintigraphy
- Laboratory tests: complete microscopical tests

Finally, we resume the benefits of osteostimulative progenitor factor therapy:

- It is a modern therapy, for the future
- It is relatively easy to apply and is not requiring very complicated instruments
- It is a dynamic method, with action in time, at distance from the moment of surgery
- It is a biological surgical method belonging to the so–called ‘ecological surgery’
- It has a wide range of indications and the preparations are relatively easy to obtain
- Clinical experience of many practitioners qualifies it as an exceptional therapeutic method
- Giving the large applicability sphere (over 55% of the casuistic), we consider that intensifying research especially interesting osteoblasts cells cultures will be particularly benefic in solving an extended range of problems of locomotory apparatus pathologies
